# Two ENU-Induced Alleles of *Atp2b2* Cause Deafness in Mice

**DOI:** 10.1371/journal.pone.0067479

**Published:** 2013-06-24

**Authors:** Marina R. Carpinelli, Michael G. Manning, Benjamin T. Kile, A. Burt Rachel

**Affiliations:** 1 Murdoch Childrens Research Institute, Royal Children’s Hospital, Parkville, Victoria, Australia; 2 The HEARing Cooperative Research Centre, University of Melbourne, Melbourne, Victoria, Australia; 3 Department of Genetics, University of Melbourne, Melbourne, Victoria, Australia; 4 Walter and Eliza Hall Institute of Medical Research, Parkville, Victoria, Australia; 5 Department of Medical Biology, University of Melbourne, Melbourne, Victoria, Australia; National Institutes of Health / NICHD, United States of America

## Abstract

Over 120 loci are known to cause inherited hearing loss in humans. The deafness gene has been identified for only half of these loci. With the aim of identifying some of the remaining deafness genes, we performed an ethylnitrosourea mutagenesis screen for deaf mice. We isolated two mutants with semi-dominant hearing loss, *Deaf11* and *Deaf13*. Both contained causative mutations in *Atp2b2*, which encodes the plasma membrane calcium ATPase 2. The *Atp2b2* ^*Deaf11*^ mutation leads to a p. I1023S substitution in the tenth transmembrane domain. The *Atp2b2* ^*Deaf13*^ mutation leads to a p. R561S substitution in the catalytic core. Mice homozygous for these mutations display profound hearing loss. Heterozygotes display mild to moderate, progressive hearing loss.

## Introduction

Inherited hearing loss affects 1 in 500 children [1]. Over 120 loci have been linked to non-syndromic hearing loss in humans but only 57 genes have been identified (http://hereditaryhearingloss.org) [2], leaving many patients without a genetic diagnosis. Development of new genetic tests for hearing loss would provide families with information such as likelihood of progression, suitability for cochlear implant, carrier status of other children and likelihood of having another child with hearing loss.

After travelling through the outer and middle ear, sound reaches the cochlea, which is divided into three fluid-filled compartments [3]. The scala vestibuli and scala tympani contain perilymph and the scala media contains endolymph [3]. Endolymph contains a high concentration of potassium ions and also contains calcium ions [3]. Hair cells project actin-filled stereocilia from the organ of Corti into the endolymph [3]. Sound waves bend the stereocilia, stretching the tip links between them and pulling open a mechanoelectrical transduction (MET) channel [3]. This allows potassium and calcium ions from the endolymph to enter the hair cell, causing the hair cell to signal to synapsing neurons of the auditory nerve [3].

We performed a mouse mutagenesis screen to isolate dominant mutations causing hearing loss. In this paper we describe the characterization of two mutants with semi-dominant hearing loss. The deafness mutations in these mice lie in *Atp2b2*, which encodes the plasma membrane calcium ATPase 2 (PMCA2). This pump is expressed in cochlear hair cells [4] and exports calcium into the endolymph (reviewed in 5). Ten previously published *Atp2b2* mutant mice display hearing loss, vestibular problems and ataxia (table 1) [6–14]. Our new alleles of *Atp2b2* identify two amino acids that are functionally important in PMCA2.

## Materials and Methods

### Mice

All mice were bred and maintained at the Walter and Eliza Hall Institute of Medical Research (WEHI). Animals were group-housed in individually ventilated micro-isolator cages (Airlaw, Smithfield, NSW, Australia) enriched with a plastic house/toy and sunflower seeds on a 12-hour light/dark cycle. Animals were fed standard Barastoc mouse chow (Ridley AgriProducts, Melbourne, VIC, Australia) and water *ad libitum*. The WEHI animal ethics committee approved this work in project numbers 2008.023 and 2011.016.

### Mutagenesis Screen

Male BALB/c mice were intraperitoneally injected with 85 mg/kg ethylnitrosourea (ENU) (Sigma-Aldrich, Castle Hill, NSW, Australia) weekly for 3 weeks as described [15]. Treated animals were rested for 12 weeks before mating with untreated BALB/c females to produce first generation (G_1_) progeny. These mice were acoustic startle response (ASR)-tested at 8 weeks of age. Animals with maximum startle amplitude below 200 arbitrary units were test-mated to determine heritability of the phenotype. Mutant strains were backcrossed for 10 generations in order to breed out irrelevant ENU-induced mutations.

### Acoustic Startle Response

ASR was measured using an SR-LAB system (San Diego Instruments, San Diego, CA, USA). Testing was conducted in the light phase of the light cycle and the testing environment was illuminated. Each mouse was restrained in a perspex chamber and acclimatized to background white noise of 70 dB SPL for 1 min. Trials were presented in pseudorandom order and separated by intervals of 3-8 sec. Mice underwent 6 background noise trials and 6 each of 85, 90, 95, 100 and 115 dB SPL 40 msec white noise pulse trials. After deleting the largest and smallest values, the average startle amplitude for each stimulus was calculated and plotted against sound intensity using Prism v 6.0b software (GraphPad Software Inc, La Jolla, CA, USA).

### Auditory Brainstem Response

Mice were anaesthetized by intraperitoneal injection of 100 mg/kg ketamine and 20 mg/kg xylazine and their eyes were moistened with lacrilube. Body temperature was maintained with a 37°C heat pad inside a custom-made faraday chamber. The faraday chamber was placed inside a sound attenuation cabinet, the Habitest isolation cubicle model H10-24A (Coulbourn Instruments, Whitehall, PA, USA). A free-field magnetic speaker (model FF1, Tucker Davis Technologies, Alachua, Florida, USA) was placed 10 cm from the left pinna and computer-generated clicks (100 µsec duration, with a spectrum of 0–50 kHz) and pure tone stimuli of 4, 8, 16 and 32 kHz were presented with maximum intensities of 100 dB SPL. Auditory brainstem responses (ABRs) were recorded differentially using subdermal needle electrodes (S06666-0, Rochester Electro-Medical, Inc., Lutz, FL, USA). These were positioned at the vertex of the skull (+ve) and on the left cheek (-ve) with a ground on the hind left leg. ABRs were averaged over 512 repetitions of the stimulus. ABR traces were analyzed to determine ABR threshold using BioSig software (Tucker Davis Technologies). The threshold was defined as the lowest intensity stimulus that reproducibly elicited an ABR.

### Mutation Identification

Exome sequencing of two *+/Deaf11* and two *+/Deaf13* mice was performed at the Australian Genome Research Facility (AGRF) using the 100803_MM9_exome_rebal_2_EZ_HX1 exome capture array (Roche Nimblegen, Madison, WI, USA), TruSeq Sample Preparation Kit (Illumina, San Diego, CA, USA) and HiSeq2000 Sequencing System (Illumina). Sequence analysis was performed by the Bioinformatics Unit of the Australian Phenomics Facility (APF) using a custom workflow to align the sequence reads to the reference genome (C57BL/6 NCBI m37), filter the raw single nucleotide variant (SNV) calls and generate a list of candidate SNVs as described [16]. Deep-sequencing datasets were deposited into the National Center for Biotechnology Information (NCBI) Sequence Read Archive (http://www.ncbi.nlm.nih.gov/sra) with the run accession numbers SRR822874, SRR822875, SRR822876 and SRR822877.

*Atp2b2* SNVs were amplified using primers MC47 and MC48 (table 2) in *Deaf11* mice and primers MC99 and MC100 in *Deaf13* mice. 25 µl PCR reactions contained 2 µl genomic DNA, 1xPCR buffer (Life Technologies, Mulgrave, VIC, Australia), 500 nM each primer, 200 nM dNTPs, 1.5 mM MgCl_2_ and 0.625 U Taq DNA polymerase (Life Technologies). Reactions were incubated at 94°C for 3 min then for 30 cycles of 94°C for 45 sec, 55°C for 30 sec and 72°C for 90 sec, with a final extension at 72°C for 10 min. PCR products were visualized by agarose gel electrophoresis.

**Table 1:** Published mouse *Atp2b2* alleles.

**Allele**	**Derivation**	**Mutation**	**Inheritance**	**Effect**	**Ref**
*dfw*	spontaneous	point	recessive	G283S	[6]
*dfw-2J*	spontaneous	deletion	semidominant	Null allele	[6]
*dfw-3J*	spontaneous	deletion	semidominant	Null allele	[7]
*Elfin*	ENU	point	semidominant	I655N	[8]
*jog*	spontaneous	insertion	recessive	↓expression	[9]
*M1Mae*	spontaneous	point	semidominant	T692K	[10]
*Obv*	ENU	point	semidominant	S877F	[11]
*tm1Ges*	gene targeting	insertion	semidominant	Null allele	[12]
*Tmy*	ENU	point	semidominant	E629K	[13]
*wri*	spontaneous	point	semidominant	E412K	[14]

Ref reference.

**Table 2:** Primers used for mutation identification.

**Primer**	**Sequence**
MC47	GACCCGCAGATAGTGATCGT
MC48	TTCCCTGGTATGGGCAGTAG
MC99	CCTCGGGGTCAAAATGATAA
MC100	GTGATGCAGACACCCACAAC

Unincorporated nucleotides and primers were removed with Exo-SAP-IT exonuclease (Affymetrix, Santa Clara, CA, USA) according to the manufacturer’s instructions. 10 µl sequencing reactions containing 3 µl PCR product, 160 nM primer, 2 µl BigDye terminator v 3.1 (Life Technologies) and 1 µl 5x buffer (Life Technologies) were incubated at 96°C for 2 min, followed by 25 cycles of 96°C for 10 sec, 50°C for 5 sec and 60°C for 4 min. Sequencing products were precipitated by addition of 75µl 200 nM MgSO_4_ in 70% ethanol. After 15 min incubation samples were centrifuged at 15,800*g* for 15 min. Pellets were washed with 70% ethanol, dried at 37°C and submitted to the AGRF for capillary separation. Sequencing electropherograms were aligned using Seqman v 10.1 software (DNASTAR, Madison, WI, USA). The NCBI protein database (http://www.ncbi.nlm.nih.gov/protein/) entry Q9R0K7.2 was used to assign domains to the PMCA2 amino acid sequence.

### Linkage Mapping

An *Atp2b2* ^*Deaf11/Deaf11*^ mouse was crossed to a C57BL/6 mouse to generate N_1_ offspring. N_1_ mice were intercrossed to produce 87 N _1_F_1_ offspring, which were ABR-tested at 8 weeks of age. Genomic DNA was extracted from liver as described [17] and genotyped for single nucleotide polymorphisms (SNPs) on chromosome 6 using the Amplifluor SNPs HT genotyping system FAM-JOE (Merck Millipore, Kilsyth, VIC, Australia) and primers listed in Table S1. DNA was vacuum-dried onto a 384 well plate. 5 µl PCR reactions containing 0.15 µM each forward primer, 2.25 µM reverse primer, 0.2 mM each dNTP (Merck Millipore), 1xFAM (Merck Millipore), 1xJOE (Merck Millipore), 1xS+ mix (Merck Millipore) and 0.05 µl titanium Taq DNA polymerase (Clontech Laboratories, Mountain View, CA, USA) were added to the plate using an epMotion 5070 robot (Eppendorf, South Pacific, North Ryde, NSW, Australia). PCR reactions were incubated at 94°C for 5 min, followed by 20 cycles of 94°C for 10 sec, 55°C for 5 sec, 72°C for 10 sec, followed by 22 cycles of 94°C for 10 sec, 55°C for 20 sec, 72°C for 40 sec, followed by a final extension at 72°C for 3 min. Fluorescence was measured with an infinite M200PRO plate reader (Tecan, Männedorf, Switzerland) using Magellan v 7.1 software (Tecan). FAM was excited at 490 nm and emission measured at 530 nm. JOE was excited at 520 nm and emission measured at 560 nm. Results were visualized and genotypes assigned using assayauditorEP.xls (Merck Millipore) and excel v 12.2.0 software (Microsoft, Redmond, WA, USA).

### Histology

All mice used for histology had previously been ABR-tested at 4 and 8 weeks of age. Mice were euthanized by intraperitoneal injection of 400 mg/kg ketamine and 80 mg/kg xylazine. After cessation of breathing, PBS was perfused through each animal via a cannula inserted into the left ventricle for 5 min, followed by 10% neutral buffered formalin for 5 min. Cochleae were dissected from the temporal bones and post-fixed for 1 hr at room temperature. Cochleae were washed in tris-buffered saline and decalcified in 10% EDTA for 5 days at 4°C with gentle rolling. Cochleae were oriented in 1% agarose in PBS in 10 mm x 10 mm x 5 mm cryomolds (Sakura Finetek, Torrance, CA, USA) and paraffin-embedded. 2 µm sections were cut parallel to the modiolus using a microtome and stained with hematoxylin and eosin (H&E). Sections were imaged with a DM1000 compound microscope (Leica Microsystems, North Ryde, Australia) and DFC450 C camera (Leica Microsystems).

### Statistical Analysis

One-way ANOVA and Dunnett’s multiple comparison test were performed using Prism 6 software (GraphPad Software Inc.).

## Results

We undertook an ENU mutagenesis screen for hearing loss in which we screened 1,462 G_1_ mice for low ASR. Control BALB/c mice consistently displayed maximum startle amplitude above 200 arbitrary units at 115 dB SPL (data not shown). Therefore, the criterion used to identify G_1_ mice for re-testing was maximum startle amplitude below 200 arbitrary units. G_1_ mice displaying a consistent low ASR were progeny-tested. This led to the isolation of 14 mutant lines with dominant, low ASR. Many of these will be described in future publications. In this paper we describe two of these mutant lines, *Deaf11* and *Deaf13*.

**Figure 1:**
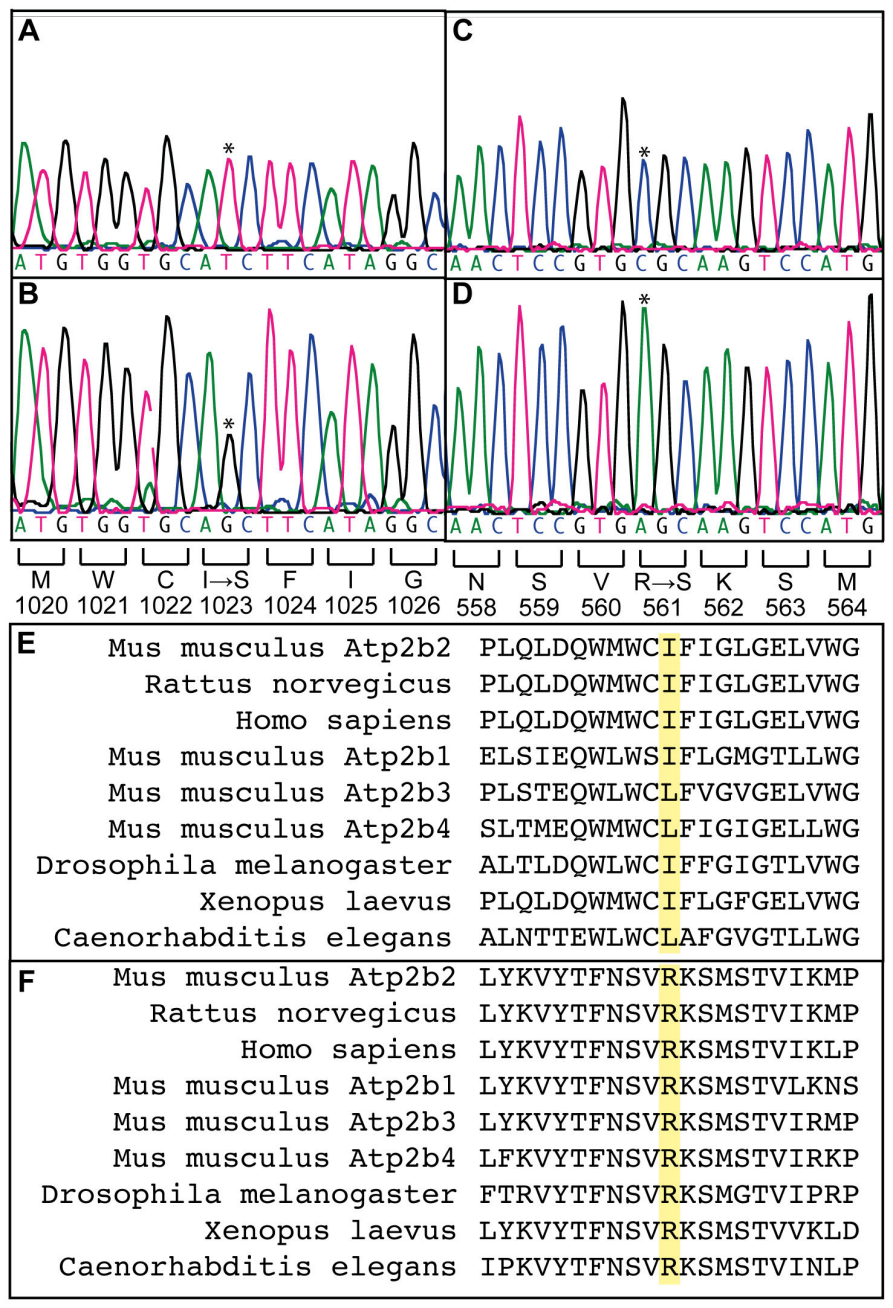
*Deaf11* and *Deaf13* mice harbor point mutations in *Atp2b2*. DNA sequence electropherograms of exon 17 in **A**) *+/+* and **B**) *Deaf11/Deaf11* mice. Results are representative of four *+/+*, 14 *+/Deaf11* and 10 *Deaf11/Deaf11* mice. DNA sequence electropherograms of exon 9 in **C**) *+/+* and **D**) *Deaf13/Deaf13* mice. Results are representative of two *+/+*, four *+/Deaf13* and four *Deaf13/Deaf13* mice. Mutation sites are marked by asterisks. Predicted effect on amino acid sequence is shown below. **E**) Amino acid sequence alignments for regions surrounding predicted *Deaf11* and **F**) Deaf 13 amino acid substitutions (yellow). Alignments were performed with Clustal Omega (http://www.ebi.ac.uk/Tools/msa/clustalo/) using protein sequences Q9R0K7.2, P11506.2, Q01814.2, NP_080758.1, NP_796210.2, AAI09173.1, NP_001033803.3, NP_001087020.1 and CAA09303.1.

Genomic DNA from two *+/Deaf11* and two *+/Deaf13* mice was exome-enriched and massively parallel sequenced. Eighty-nine percent of the consensus coding sequence (CCDS) exome was sequenced at least 4-fold (Table S2). The average depth of sequencing was between 37 and 69-fold. Between 25 and 1459 SNVs were identified in each sample. Visual comparison revealed that all samples contained a SNV in *Atp2b2*. These SNVs were confirmed by Sanger DNA sequencing. In *Deaf11* mice a c.3068T>G mutation in exon 17 encodes a p. I1023S substitution in the tenth transmembrane domain of PMCA2 (Figure 1A-B). In *Deaf13* mice a c.1681C>A mutation in exon 9 encodes a p. R561S substitution in the catalytic core of the PMCA2 pump (Figure 1C-D) [18]. Amino acid alignments indicate that R561 is conserved through evolution while I1023 is conserved or substituted with the similar amino acid, leucine (Figure 1E-F). This indicates that these amino acids are functionally important in PMCA2. As these SNVs are not present in the WEHI BALB/c mouse strain (data not shown), they are likely to be ENU-induced mutations.

**Figure 2:**
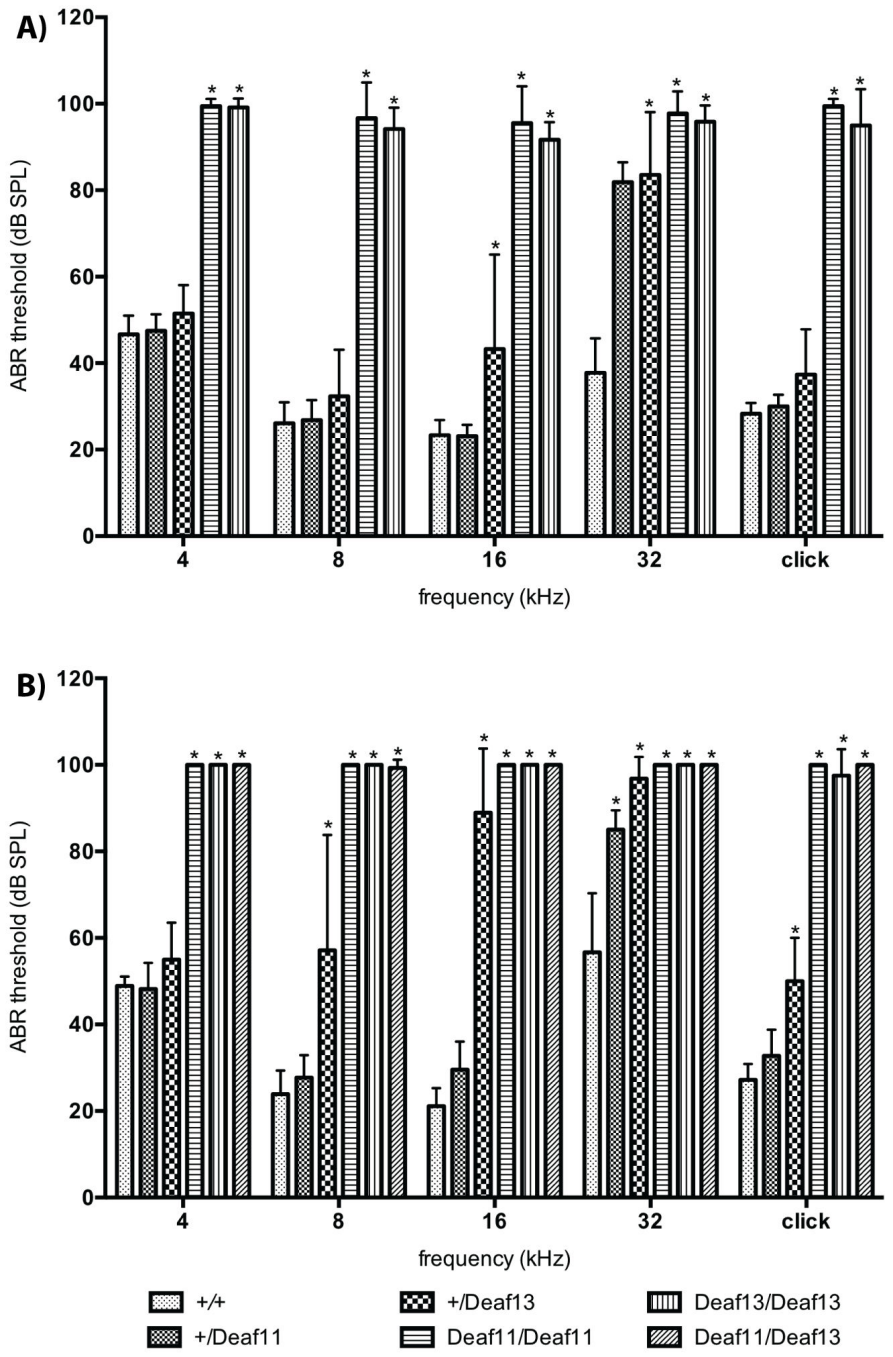
*Deaf11* and *Deaf13* mice display hearing loss. Mean ABR thresholds at **A**) 4 and **B**) 8 weeks of age; *Atp2b2* ^*+/+*^ n=3 female (F), 6 male (M) at 4 wks, n = 4F, 5M at 8 wks, *Atp2b2* ^*+/Deaf11*^ n = 6F, 2M at 4 wks, n = 7F, 4M at 8 wks, *Atp2b2* ^*+/Deaf13*^ n = 7F, 10M at 4 wks, n = 5F, 9M at 8 wks, *Atp2b2* ^*Deaf11/Deaf11*^ n = 3F, 6M at 4 wks, n = 2F and 5M at 8 wks, *Atp2b2* ^*Deaf13/Deaf13*^ n = 2F, 4M, *Atp2b2* ^*Deaf11/Deaf13*^ n = 4F, 3M; * p<0.01 versus *Atp2b2* ^*+/+*^; Error bars show standard deviation.

*Deaf11* and *Deaf13* mice displayed normal body weight at 8 weeks of age (data not shown). We performed ABR tests to determine whether the low ASR was caused by hearing loss. ABR thresholds at 4, 8, 16 and 32 kHz and for broad frequency click stimuli were determined at 4 and 8 weeks of age in *Atp2b2* ^*+/+*^, *Atp2b2* ^*+/Deaf11*^, *Atp2b2* ^*+/Deaf13*^, *Atp2b2* ^*Deaf11/Deaf11*^, *Atp2b2* ^*Deaf13/Deaf13*^ and *Atp2b2* ^*Deaf11/Deaf13*^ mice. One-way ANOVA showed a statistically significant difference between mean thresholds at all frequencies tested (Table S3). Dunnett’s test was then used to compare mean thresholds to those of *Atp2b2* ^*+/+*^ mice, with a multiplicity-adjusted p-value of less than 0.01 taken as statistically significant. *Atp2b2* ^*Deaf11/Deaf11*^ and *Atp2b2* ^*Deaf13/Deaf13*^ mice had profound hearing loss by 4 weeks of age. At this age, *Atp2b2* ^*+/Deaf13*^ mice had high frequency hearing loss (Figure 2A). By 8 weeks of age, *Atp2b2* ^*+/Deaf11*^ and *Atp2b2* ^*+/Deaf13*^ mice displayed hearing loss at middle and high frequencies. The profound hearing loss observed in *Atp2b2* ^*Deaf11/Deaf11*^ and *Atp2b2* ^*Deaf13/Deaf13*^ mice also occurred in *Atp2b2* ^*Deaf11/Deaf13*^ mice (Figure 2B). The lack of complementation between *Deaf11* and *Deaf13* indicated that the causative mutations were allelic. Gender had no statistically significant effect on ABR thresholds.

**Figure 3:**
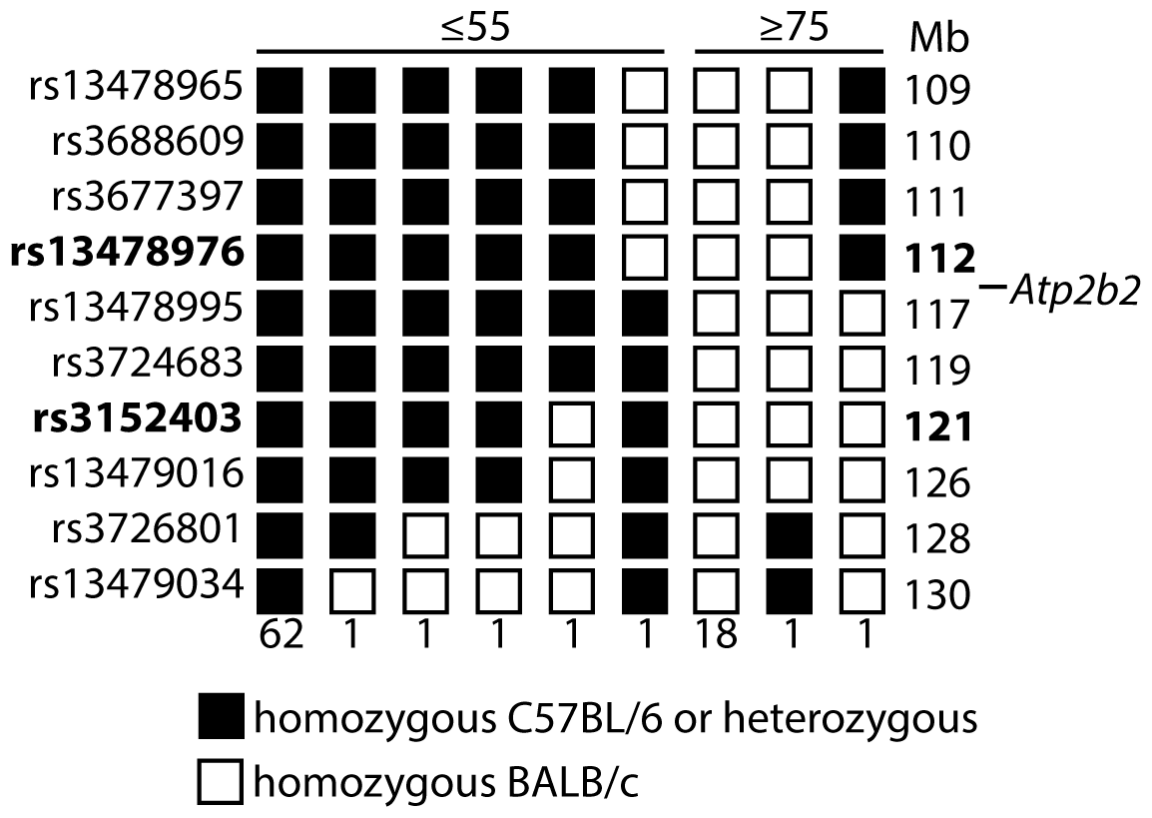
The *Deaf11* mutation is linked to chromosome 6. A *Deaf11/Deaf11* BALB/c mouse was crossed to a *+/+* C57BL/6 mouse and the offspring intercrossed to generate 87 N _1_F_1_ mice. Mice were divided into those with a click ABR threshold ≤55 dB SPL on left (*+/+* or *+/Deaf11*) and those with a threshold ≥75 dB SPL on right (*Deaf11/Deaf11*). All mice were genotyped for SNPs on chromosome 6. SNP names and locations are listed on the left and right respectively (assembly GpCm38, database SNP, http://www.ncbi.nlm.nih.gov/projects/SNP/). The numbers of mice displaying each haplotype are listed below. Informative recombinations indicate that the *Deaf11* causative mutation lies between rs13478976 at 112 Mb and rs3152403 at 121 Mb. *Atp2b2* is located at 113 Mb.

These *Atp2b2* mutations may have been causative of hearing loss or irrelevant. As each G_1_ mouse harbors about 43 penetrant ENU-induced mutations [19], or one every 63 Mb, irrelevant mutations are unlikely to be genetically linked to hearing loss. We performed meiotic mapping in order to determine whether the *Atp2b2* ^*Deaf11*^ mutation was genetically linked to hearing loss (Figure 3). We crossed an *Atp2b2* ^*Deaf11/Deaf11*^ BALB/c mouse to an *Atp2b2* ^*+/+*^ C57BL/6 mouse. We chose this strain partly because C57BL/6 mice have similar ABR thresholds to BALB/c mice [20]. Offspring were intercrossed to generate 87 N _1_F_1_ mice. These were ABR-tested and genotyped for SNPs between 109 and 130 Mb on chromosome 6. Informative recombinations allowed us to localise the *Deaf11* mutation to between the SNPs rs13478976 at 112 Mb and rs3152403 at 121 Mb. This region encompasses the *Atp2b2* gene at 113 Mb, indicating that the *Atp2b2* ^*Deaf11*^ mutation is causative of the hearing loss in *Deaf11* mice. As the *Deaf11* and *Deaf13* mutations are allelic, we can infer that the *Atp2b2* ^*Deaf13*^ mutation is causative of hearing loss in *Deaf13* mice.

**Figure 4:**
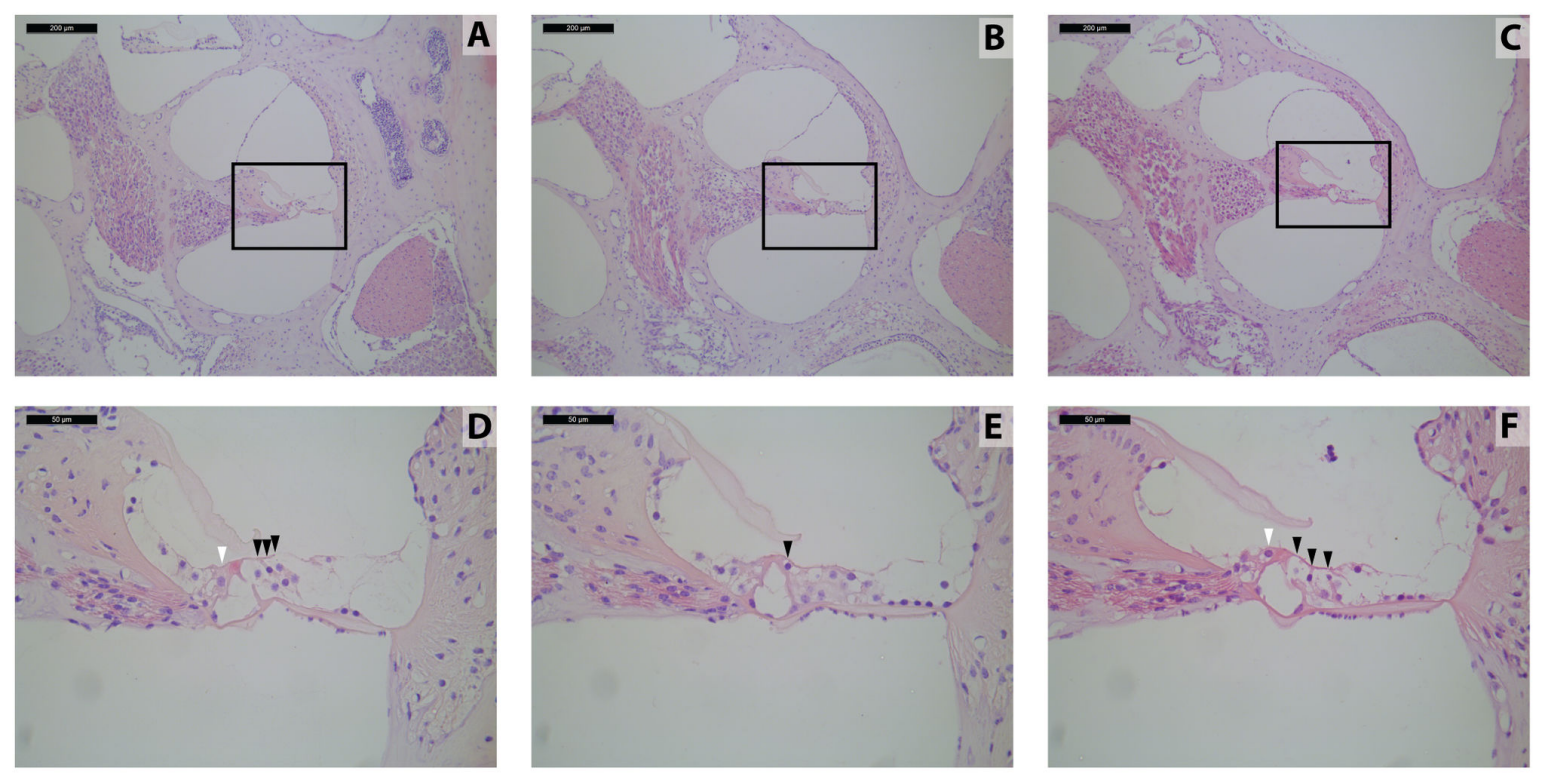
*Deaf11* and *Deaf13* cochlear hair cells are morphologically abnormal. Middle cochlear turn sections at 8 weeks of age; Upper panels 100x magnification, scale bar 200 µm; Lower panels 400x magnification, scale bar 50 µm; **A**, **D**) *Atp2b2* ^*+/+*^ **B**, **E**) *Atp2b2* ^*Deaf11/Deaf11*^ **C**, **F**) *Atp2b2* ^*Deaf13/Deaf13*^; White arrowheads point to inner hair cells; Black arrowheads point to outer hair cells; Each section is representative of 4 cochleae.

PMCA2 is expressed in cochlear hair cells [4] and facilitates export of calcium into endolymph [5]. Although the primary role of PMCA2 is not in hair cell survival, hair cells could die as a secondary effect of insufficient PMCA2 activity. We examined this by collecting mid-modiolar cochlear sections from *Atp2b2* ^*+/+*^, *Atp2b2* ^*Deaf11/Deaf11*^ and *Atp2b2* ^*Deaf13/Deaf13*^ mice at 8 weeks of age. As these mice were ABR-tested at 4 and 8 weeks of age, we cannot exclude the possibility that their cochlear hair cell survival was affected by this test. Three outer hair cells and one inner hair cell were apparent in the middle turn of *Atp2b2* ^*+/+*^ cochlea. The *Atp2b2* ^*Deaf11/Deaf11*^ and *Atp2b2* ^*Deaf13/Deaf13*^ hair cells had abnormal morphology indicative of degeneration (Figure 4). These results suggest that the loss of PMCA2 activity may lead to degeneration of hair cells in *Atp2b2* ^*Deaf11/Deaf11*^ and *Atp2b2* ^*Deaf13/Deaf13*^ mice.

## Discussion

This study demonstrates that isoleucine 1023 and arginine 561 are functionally important in PMCA2. Isoleucine 1023 lies in the 10^th^ transmembrane domain. In *Deaf11* mice this hydrophobic amino acid has been substituted with the polar amino acid serine. This may disrupt insertion of PMCA2 into the cell membrane. Arginine 561 lies in the catalytic core of the pump. In *Deaf13* mice this basic amino acid has been replaced with the polar amino acid serine. This may disrupt the enzymatic activity of PMCA2. There are now eight published mouse mutants with amino acid substitutions disrupting PMCA2 activity (table 1). These will help to elucidate the molecular mechanism whereby PMCA2 exports calcium from the cell.

A number of observations suggest that the *Deaf11* and *Deaf13* alleles are hypomorphic. *Atp2b2* ^*Deaf11/Deaf11*^ and *Atp2b2* ^*Deaf13/Deaf13*^ mice do not display ataxia or stunted growth, as observed in mice homozygous for the null allele [12]. Furthermore, *Atp2b2* ^*Deaf11/+*^ and *Atp2b2* ^*Deaf13/+*^ mice have milder hearing loss than mice heterozygous for the null allele [12]. The *Deaf11* and *Deaf13* mice may prove useful for those wishing to study milder forms of *Atp2b2*-induced hearing loss.

The mechanism whereby decreased PMCA2 activity causes hearing loss in *Deaf11*, *Deaf13* and other *Atp2b2* mutant mice is unknown. *Atp2b2* ^*dfw-2J/dfw-2J*^ mice display an abnormally low calcium concentration in endolymph [21]. This corresponds with low endocochlear potential, which would be expected to disrupt mechanoelectrical transduction [21]. Low calcium concentration in endolymph could also interfere with other calcium-dependent processes in hearing. For example, tip links are composed of the calcium-binding proteins cadherin 23 and protocadherin 15 [22] and break in the absence of calcium [23].

Middle turn cochlear hair cells in *Atp2b2* ^*Deaf11/Deaf11*^ and *Atp2b2* ^*Deaf13/Deaf13*^ mice have abnormal morphology at 8 weeks of age. Cochlear hair cells degenerate in other *Atp2b2* mutant mice [8,11,12,14]. High intracellular calcium levels could induce apoptosis in these hair cells. In support of this hypothesis, PMCA2 delays apoptosis in hair cells with calcium overload secondary to a constitutively open TRPML3 channel [24].

The *Cdh23* ^*753A*^ allele accelerates hearing loss in *Atp2b2* ^*dfw-2J/+*^ mice [25,26]. C57BL/6J and BALB/cByJ mice carry the susceptible *Cdh23* ^*753A*^ allele [26]. BALB/cJ mice are likely to also carry the *Cdh23* ^*753A*^ allele as they are closely related to BALB/cByJ [27]. Hearing loss may not be penetrant in *Atp2b2* ^*Deaf11/+*^ and *Atp2b2* ^*Deaf13/+*^ mice on genetic backgrounds carrying the *Cdh23* ^*753G*^ resistant allele, such as CBA/CaJ and C3HeB/FeJ [26]. In a human family, an *Atp2b2* mutation worsens hearing loss in patients homozygous for a *Cdh23* mutation [28]. In another family, a patient with hearing loss is a compound heterozygote for *Cdh23* and *Atp2b2* mutations, while three other family members with normal hearing are single heterozygotes [18]. There is no evidence linking *Atp2b2* with monogenic hearing loss in humans. It is located on chromosome 3p25, 3 [29], a region to which no syndromic or non-syndromic hearing loss loci have been mapped [30].

## Supporting Information

Table S1 **Primers used in SNP genotyping.**
(DOCX)

Table S2 **Massively parallel sequencing results.**
(DOCX)

Table S3 **One way ANOVA results for ABR thresholds.**
(DOCX)
