# Correction: Two ENU-Induced Alleles of *Atp2b2* Cause Deafness in Mice

**DOI:** 10.1371/annotation/f605bb2c-37b6-4fa7-a213-324df679c464

**Published:** 2013-07-09

**Authors:** Marina R. Carpinelli, Michael G. Manning, Benjamin T. Kile, Rachel A. Burt

The name of the fourth author was given incorrectly. The correct name is: Rachel A. Burt. The correct citation is: Carpinelli MR, Manning MG, Kile BT, Burt RA (2013) Two ENU-Induced Alleles of *Atp2b2* Cause Deafness in Mice. PLoS ONE 8(6): e67479. doi:10.1371/journal.pone.0067479. 

In addition, affiliations for the fourth author were missing. Rachel A. Burt is not only affiliated with institutions 1, Murdoch Childrens Research Institute, Royal Children’s Hospital, Parkville, Victoria, Australia and 4, Walter and Eliza Hall Institute of Medical Research, Parkville, Victoria, Australia, but also institutions 2, The HEARing Cooperative Research Centre, University of Melbourne, Melbourne, Victoria, Australia, and 3, Department of Genetics, University of Melbourne, Melbourne, Victoria, Australia. 

