# Supplementary material for: Two ENU-Induced Alleles of Atp2b2 Cause Deafness in Mice
Source: PLoS One. 2013 Jun 24;8(6):e67479. doi: 10.1371/journal.pone.0067479 (PMC3691321; doi:10.1371/journal.pone.0067479)
Supplement: Table S1 — Primers used in SNP genotyping. (DOCX) [file pone.0067479.s001.docx]

| **Primer** | **Sequence** |
| --- | --- |
| rs13478965-F1 | GAAGGTCGGAGTCAACGGATTTCACATCCAGGATTTCTTTCAATGC |
| rs13478965-F2 | GAAGGTGACCAAGTTCATGCTTCACATCCAGGATTTCTTTCAATGT |
| rs13478965-R | ACTGCCTCTCTGGTGTGATTT |
| rs3688609-F1 | GAAGGTGACCAAGTTCATGCTCATCACTTATGCACATTTTTAGC |
| rs3688609-F2 | GAAGGTCGGAGTCAACGGATTCTCATCACTTATGCACATTTTTAGGA |
| rs3688609-R | CCACTCTACGAGTTCCATTCAA |
| rs3677397-F1 | GAAGGTCGGAGTCAACGGATTGAGGAAGGATATACAAAGTATCATGAGTAGAAAT |
| rs3677397-F2 | GAAGGTGACCAAGTTCATGCTGAGGAAGGATATACAAAGTATCATGAGTAGAAAC |
| rs3677397-R | ATCATCAGTTTTAACAGAAGATCATCAT |
| rs13478976-F1 | GAAGGTCGGAGTCAACGGATTCTCTCTTCCTCTTCTTATACAAGGCT |
| rs13478976-F2 | GAAGGTGACCAAGTTCATGCTTCTCTTCCTCTTCTTATACAAGGCG |
| rs13478976-R | AAGTTTGGAGAATGGGAACCCA |
| rs13478995-F1 | GAAGGTCGGAGTCAACGGATTTTTCTCTTCTACCACAGGCTGG |
| rs13478995-F2 | GAAGGTGACCAAGTTCATGCTTTTCTCTTCTACCACAGGCTGC |
| rs13478995-R | TGCTACCTAAGACCAGGGAA |
| rs3724683-F1 | GAAGGTCGGAGTCAACGGATTGAAGGTGCAGCTGAGACACCA |
| rs3724683-F2 | GAAGGTGACCAAGTTCATGCTAAGGTGCAGCTGAGACACCG |
| rs3724683-R | GCTTTCTCCCTCCAGCTT |
| rs3152403-F1 | GAAGGTGACCAAGTTCATGCTGCCTGTTTAACTGTCTGTTCTTCAAT |
| rs3152403-F2 | GAAGGTCGGAGTCAACGGATTCCTGTTTAACTGTCTGTTCTTCAAC |
| rs3152403-R | TGTCACTGACCAGACGACAATA |
| rs13479016-F1 | GAAGGTCGGAGTCAACGGATTTTTGCCAATGGCACCGTAAC |
| rs13479016-F2 | GAAGGTGACCAAGTTCATGCTGTTTGCCAATGGCACCGTAAT |
| rs13479016-R | GACACCTGAGCTTCTGACTT |
| rs3726801-F1 | GAAGGTCGGAGTCAACGGATTTCCCTTAAGAACATGGCAAAGACG |
| rs3726801-F2 | GAAGGTGACCAAGTTCATGCTATCCCTTAAGAACATGGCAAAGACA |
| rs3726801-R | AGGCTATGTGATGGATCACAGTA |
| rs13479034-F1 | GAAGGTCGGAGTCAACGGATTCTGTGTTTACCCCCAAATACTGAGTT |
| rs13479034-F2 | GAAGGTGACCAAGTTCATGCTTCCTGTGTTTACCCCCAAATACTGAGTA |
| rs13479034-R | CCACACCTACATTCTGGAAAAA |
