# Supplementary material for: Two ENU-Induced Alleles of Atp2b2 Cause Deafness in Mice
Source: PLoS One. 2013 Jun 24;8(6):e67479. doi: 10.1371/journal.pone.0067479 (PMC3691321; doi:10.1371/journal.pone.0067479)
Supplement: Table S2 — Massively parallel sequencing results. (DOCX) [file pone.0067479.s002.docx]

| **Mouse** | **Coverage (%)** | **Depth (fold)** | **Number of SNVs** |
| --- | --- | --- | --- |
| Deaf11.G021 | 89.5 | 69 | 25 |
| Deaf11.G022 | 89.2 | 40 | 143 |
| Deaf13.F001 | 88.7 | 37 | 1026 |
| Deaf13.F002 | 88.8 | 43 | 1459 |
