# Supplementary material for: Two ENU-Induced Alleles of Atp2b2 Cause Deafness in Mice
Source: PLoS One. 2013 Jun 24;8(6):e67479. doi: 10.1371/journal.pone.0067479 (PMC3691321; doi:10.1371/journal.pone.0067479)
Supplement: Table S3 — One way ANOVA results for ABR thresholds. (DOCX) [file pone.0067479.s003.docx]

| **Age** | **Frequency** | **F value** | **Degrees of freedom** | **p-value** |
| --- | --- | --- | --- | --- |
| 4 weeks | click | 216.6 | 48 | < 0.0001 |
| 4 weeks | 4 kHz | 294.4 | 48 | < 0.0001 |
| 4 weeks | 8 kHz | 176.8 | 48 | < 0.0001 |
| 4 weeks | 16 kHz | 54.53 | 48 | < 0.0001 |
| 4 weeks | 32 kHz | 94.34 | 48 | < 0.0001 |
| 8 weeks | click | 245.0 | 53 | < 0.0001 |
| 8 weeks | 4 kHz | 215.5 | 53 | < 0.0001 |
| 8 weeks | 8 kHz | 53.88 | 53 | < 0.0001 |
| 8 weeks | 16 kHz | 175.5 | 53 | < 0.0001 |
| 8 weeks | 32 kHz | 60.77 | 53 | < 0.0001 |
